# Translational Barriers to Pharmaceutical Excipient Readiness in Marine-Derived Polymers

**DOI:** 10.3390/polym18101179

**Published:** 2026-05-11

**Authors:** Yedi Herdiana, Syed Mahmood, Eli Halimah, Ferry Ferdiansyah Sofian

**Affiliations:** 1 Department of Pharmaceutics and Pharmaceutical Technology, Faculty of Pharmacy, Universitas Padjadjaran, Sumedang 45363, West Java, Indonesia; y.herdiana@unpad.ac.id; 2 Department of Pharmaceutical Technology, Faculty of Pharmacy, Universiti Malaya, Kuala Lumpur 50603, Malaysia; syedmahmood@um.edu.my; 3 Faculty of Medicine, Universiti Malaya Research Centre for Biopharmaceuticals and Advanced Therapeutics (UBAT), Universiti Malaya, Kuala Lumpur 50603, Malaysia; 4 Centre of Advanced Materials (CAM), Faculty of Engineering, Universiti Malaya, Kuala Lumpur 50603, Malaysia; 5 Faculty of Pharmaceutical Sciences, Chulalongkorn University, Pathum Wan, Bangkok 10330, Thailand; 6 Department of Pharmacology and Clinical Pharmacy, Faculty of Pharmacy, Universitas Padjadjaran, Sumedang 45363, West Java, Indonesia; eli.halimah@unpad.ac.id; 7 Department of Pharmaceutical Biology, Faculty of Pharmacy, Padjadjaran University, Sumedang 45363, West Java, Indonesia

**Keywords:** marine-derived polymers, chitosan, alginate, carrageenan, pharmaceutical excipients, critical material attributes

## Abstract

Marine polymers have attracted a lot of attention as potential alternatives to the traditional animal-derived polymers in pharmaceutical formulation since they are abundant, biocompatible, and versatile in functionality. However, the presence of these materials in dosage-form studies, often in support of proof-of-concept trials, does not mean they are ready to apply as excipients routinely. This review critically evaluates the reasons why three of the most highly researched marine-derived polymers, chitosan, alginate, and carrageenan, continue to encounter significant translational barriers in pharmaceutical excipient development. All three polymers have been demonstrated to have clear pharmaceutical utility; however, their behavior is highly dependent on source, structure, processing history and formulation context. Chitosan explains why functional benefits may be compromised by responses to material requirements; alginate explains why apparent proximity to use may not remove composition-related variability; and carrageenan explains that even seemingly simple rheological functions may be very context-dependent. All of this points to the fact that the major hurdle lies not in the lack of potential, but in the difficulty of achieving the required degree of control, reproducibility, and manufacturability in order to make the reliable use of excipients possible. Future progress in this field will likely require a shift from descriptive exploration toward readiness-focused evidence, including demonstrated control over material attributes, reproducible performance, and feasible qualification pathways.

## 1. Introduction

The development of pharmaceutical science would not only rely on the innovation of the active pharmaceutical ingredient, but also on the establishment of the excipients that facilitate stable, manufacturable and functionally effective dosage forms. Polymeric excipients, in this case, are key factors in the pharmaceutical formulation; they assist in the regulation of drug release, dosage-form integrity, bioavailability improvement, and processability of a wide variety of delivery systems [1,2]. Several pharmaceutical and biomedical excipients are derived from animal sources, including gelatin, collagen, lactose, shellac, certain lipid-based excipients, and magnesium stearate from animal or mixed origins. Among these, gelatin and collagen are particularly relevant polymeric materials because they are widely used in capsules, films, wound dressings, scaffolds, and tissue-engineering platforms. Their continued use is supported by their functional performance, long history of industrial application, manufacturing familiarity, and established quality assurance systems [3,4].

At the same time, use of animal-derived materials gives rise to concerns about safety of the source, biological variability, ethical acceptability and religious compliance. In pharmaceutical contexts, sources of excipients in pharmaceuticals are also relevant to Muslim and Jewish consumers, as well as those pursuing vegan or vegetarian diets or avoiding animal-derived ingredients for ethical concerns [3,5]. Therefore, the search for alternative excipients should not be limited to replacing natural materials with synthetic polymers. Another important strategy is to replace animal-derived natural excipients with natural materials from non-animal sources, such as marine- or plant-derived polymers, focusing on aspects such as retaining properties of biocompatibility and biodegradability while increasing acceptability based on origin or supply-chain considerations. This perspective is also reflected in the newer literature on halal pharmaceuticals and alternative capsule shells where carrageenan, agar, starch and other hydrocolloids are presented not as universal replacements but rather as replacement candidates based on specific functions [6,7].

Replacing animal-derived materials, however, is not simply a matter of identifying alternative sources. Established excipients remain widely used because they provide formulation utility, industrial processability, supply continuity, predictable quality behavior, and regulatory familiarity [5,8]. Gelatin, for example, is valued for its gel-forming and film-forming properties, thermoreversible behavior, and practical utility in delivery systems and capsule-related applications [9,10]. Collagen retains an important position in biomaterials and regenerative applications because its structure and bioactive motifs support cell interaction and tissue-relevant performance [11]. Therefore, alternative materials must be evaluated not only for their origin or individual functional advantages, but also for their ability to provide robust, reproducible, manufacturable, and qualification-ready performance under realistic pharmaceutical conditions [12,13].

Plant-derived polymers such as cellulose derivatives, starch, pectin, guar gum, and plant mucilage have already demonstrated considerable value as pharmaceutical excipients. However, marine-derived polymers provide complementary functional properties that are not always easily replicated by terrestrial plant materials. Chitosan offers cationic and mucoadhesive behavior, alginate provides mild ionotropic gelation with divalent cations, and carrageenan contributes sulfated gel-forming and viscosity-enhancing characteristics. These properties make marine-derived polymers attractive candidates for drug delivery systems, hydrogels, wound dressings, films, beads, matrices, encapsulation systems, and other pharmaceutical platforms [14,15].

Marine-derived polymers are of interest as a wide range of materials can be derived from marine biomass. Chitosan, alginate, and carrageenan are widely mentioned in the literature of pharmaceutical and biomaterial studies and used for nanoparticles, hydrogels, films, beads, matrices, encapsulation systems, and other dosage-form platforms [14]. However, the mere presence of articles on a material does not indicate its readiness for routine pharmaceutical use. Its value in pharmaceutical development depends on its function as a laboratory raw ingredient, as well as on critical material attributes, batch-to-batch consistency, and robust impurity-management systems. These factors must align with performance guidelines to produce acceptable formulations while also supporting manufacturability and clear qualification pathways. This distinction is important because conventional materials such as gelatin and collagen already hold advantages in established supply chains, processing familiarity, accumulated safety experience, and regulatory confidence [5,16].

The central challenge for marine-derived polymers, therefore, lies not in the absence of candidate materials but in the difficulty of translating biologically variable raw materials into excipients with sufficiently controlled quality and performance [12,17]. Source heterogeneity, extraction–purification complexity, variability in molecular and compositional features, impurity burden, context-dependent formulation behavior, scale-up constraints, and qualification demands all contribute to this translational gap [14,18]. From a translational perspective, the central task is not merely to discover marine-derived polymers with useful functions, but to develop them into excipients with the degree of standardization, reproducibility, and qualification-readiness that has made gelatin industrially dependable [12,16].

However, the need for alternatives does not make substitution easier; it makes rigorous evaluation more necessary. Alternative materials fail not only when they do not work, but also when they cannot meet the broader standards that conventional excipients have made routine in pharmaceutical development. In practice, excipient acceptance depends not only on origin or conceptual promise, but also on whether a material can repeatedly satisfy expectations for performance, consistency, processability, and qualification under realistic conditions [12,14]. These benchmarks provide the basis for the readiness-oriented appraisal used in this review, where chitosan, alginate, and carrageenan are examined as candidate excipients rather than as inherently superior alternatives.

Accordingly, this critical narrative review uses chitosan, alginate, and carrageenan as model cases because they are among the most established marine-derived polymers in the pharmaceutical and biomaterials literature, making them suitable for a readiness-oriented appraisal. Rather than attempting to catalog all marine biomaterials, the review prioritizes the conceptually relevant literature on source and compositional variability, extraction–purification burden, control of critical material attributes, safety and impurity burden, formulation-specific performance, and scale-up or qualification-related barriers, with emphasis on translational relevance to pharmaceutical use. By shifting the discussion from marine polymer promise to excipient readiness, this review argues that future development should be judged less by novelty or abundance alone than by the extent to which a material can realistically meet the technical, manufacturing, and qualification demands of pharmaceutical application.

## 2. Marine-Derived Polymers as Test Cases, Not Proof of Readiness

Marine-derived polymers are discussed in this section as readiness-oriented test cases rather than as proof that natural-to-natural substitution is already pharmaceutically established. Chitosan, alginate, and carrageenan were selected because they are among the most frequently investigated marine-derived polymers in pharmaceutical and biomaterial research, yet each still illustrates important barriers between laboratory functionality and excipient readiness [19]. Their value in this review therefore lies not in presenting them as universal substitutes for gelatin, collagen, or other established excipients, but in using them to examine how polymer-specific attributes, formulation context, impurity control, manufacturability, and safety requirements determine the feasibility of pharmaceutical translation.

### 2.1. Basis for Selection

Although marine organisms contain a wide range of biomacromolecules, pharmaceutical and biomedical research has focused mainly on a limited number of representative materials. These include chitosan from crustacean-derived chitin, alginate from brown seaweed, carrageenan from red seaweed, and selected materials such as marine collagen and glycosaminoglycans [20,21]. The choice of chitosan, alginate and carrageenan in this review is hence pragmatic or appraisal-based rather than taxonomic. These three materials were selected for their relative commercial maturity, abundant formulation literature, and frequent occurrence in the pharmaceutical literature, thus making them pertinent examples for a readiness-oriented assessment [21,22].

A review concentrating on excipient preparedness does not call for the widest-possible catalog of marine candidates; it requires materials mature enough to show where translation begins to break down. If even polymers that have been widely studied continue to show repeated problems with consistency of source and control of critical material attributes, formulation reliability, and qualification feasibility, then the central problem is unlikely to be solved simply by adding another list of candidate materials [21,22]. Their value as test cases lies in showing that the challenge is not merely discovering marine-derived polymers with useful functions, but converting biologically variable and context-sensitive materials into reproducible pharmaceutical excipients.

### 2.2. Chitosan

#### 2.2.1. Source, Extraction, Purification, and Critical Material Attributes

Chitosan is one of the most intensively studied marine-derived polymers in pharmaceutical research, largely because of its cationic character and the formulation opportunities that arise from it. Its positive charge under acidic conditions supports electrostatic interaction with negatively charged biological surfaces and polymers, making it relevant to mucoadhesion, polyelectrolyte complexation, and particle formation [23,24]. These properties help explain why chitosan is repeatedly explored in drug delivery systems, especially when prolonged residence time, surface interaction, or mild particle fabrication is desirable.

Chitosan also illustrates how strong formulation utility can coexist with weak standardization. Its performance depends strongly on pH, molecular weight, degree of deacetylation, and charge-related structural features. These attributes determine solubility, interaction strength, gelation behavior, and particle-forming ability [25]. Thus, the key issue is not whether chitosan can provide useful formulation functions, but whether those functions can be reproduced consistently across sources, processes, and batches.

Extraction and purification conditions are central to chitosan quality. Demineralization, deproteinization, deacetylation, drying, and depolymerization can alter molecular weight distribution, residual mineral content, residual proteins, viscosity, and charge-related behavior. These parameters influence whether a chitosan sample can provide comparable pharmaceutical performance across sources, studies, manufacturing processes, and production batches [26,27]. Therefore, chitosan’s translational challenge lies not in the absence of useful function, but in ensuring that such function can be delivered with sufficient reproducibility to support excipient readiness [26].

#### 2.2.2. Pharmaceutical Applications

The pharmaceutical relevance of chitosan is mainly associated with its cationic, mucoadhesive, biodegradable, antimicrobial, and film-forming properties. In drug delivery, chitosan has been widely explored for nanoparticle systems, especially when mild fabrication, electrostatic complexation, residence-time extension, and interaction with biological membranes are desired [28]. Chitosan nanoparticles may be used to protect labile drugs, support mucosal delivery, improve local residence time, and enable interaction with negatively charged mucosal or cellular surfaces [29].

Chitosan is also relevant to mucoadhesive drug delivery systems, including nasal, buccal, ocular, vaginal, and gastrointestinal formulations, because its protonated amino groups can interact with mucin and epithelial surfaces [29]. In wound healing, chitosan-based films, hydrogels, and dressings are attractive because of their biodegradability, moisture-retention capacity, hemostatic potential, and reported antimicrobial activity [30]. Chitosan has also been investigated in antimicrobial films, tissue-engineering scaffolds, and polyelectrolyte complexes with anionic polymers such as alginate or carrageenan [31].

#### 2.2.3. Readiness Appraisal

Overall, chitosan can be considered a promising but highly grade-dependent excipient candidate. Its readiness is stronger in topical, wound-contact, mucoadhesive, and particulate drug delivery systems than in applications requiring broad interchangeability [26]. The main readiness barrier is the difficulty of ensuring that the same nominal material provides consistent performance across sources, processes, and grades. Thus, chitosan’s pharmaceutical value is most defensible in application-specific systems where molecular weight, degree of deacetylation, viscosity, impurity burden, and pH-dependent behavior are explicitly defined and controlled [32].

### 2.3. Alginate

#### 2.3.1. Source, Extraction, Purification, and Critical Material Attributes

Alginate is often considered one of the marine-derived polymers closer to practical pharmaceutical and biomedical use because of its relatively simple and mild ionotropic gelation behavior, particularly with calcium ions [33,34]. For hydrogels, beads, encapsulation systems, and formulations with sensitive biological components, this has made alginate particularly appealing. In comparison to many other natural polymers, alginate often looks closer to translation since its gel-forming ability can be easily demonstrated and is frequently compatible with mild processing conditions.

Apparent proximity to the application should not be confused with general preparedness. The performance of alginate still depends strongly on molecular weight, mannuronate/guluronate composition, block distribution, and the ionic environment of the system. These compositional and structural variables govern gel strength, matrix stability, and behavior under formulation conditions [35,36]. Alginate therefore illustrates a subtler readiness problem than chitosan: not limited usefulness, but a high dependence on composition-sensitive behavior that can complicate normalization as a consistently behaving excipient.

Of the three described polymers, alginate perhaps best demonstrates practical formulation maturity across selected applications. That quality makes it especially instructive. Its example indicates that clarity of application success does not mean that appraisal of readiness can be neglected, as wide use of excipients is still contingent upon control of material-specific variability at a level congruous with reproducible pharmaceutical performance.

Extraction and purification can affect alginate molecular weight, ion content, ash level, residual proteins, polyphenolic contaminants, microbial burden, and trace metal contamination. These issues are particularly important for wound-contact, cell-encapsulation, parenteral-adjacent, or tissue-engineering applications, where impurity and endotoxin control may become critical [37]. Thus, alginate illustrates a subtler readiness problem than chitosan: its usefulness is not limited, but its formulation performance depends strongly on composition-sensitive behavior that can complicate standardization as a consistently performing excipient [38].

#### 2.3.2. Pharmaceutical Applications

Alginate has broad pharmaceutical relevance because of its ionotropic gelation, biocompatibility, mild processing conditions, and ability to form hydrogels, beads, and encapsulation matrices. Calcium-crosslinked alginate beads have been investigated for oral delivery, multiparticulate systems, gastroretentive systems, and protection of sensitive compounds such as enzymes, peptides, probiotics, or living microorganisms. Alginate hydrogels are also relevant for wound dressings because they can absorb exudate, maintain a moist environment, and form soft gel-like matrices upon contact with wound fluid [37,39].

In addition, alginate is frequently used in microencapsulation and cell encapsulation because gel formation can occur under relatively mild aqueous conditions. This makes alginate attractive for sensitive biological components, including cells, proteins, peptides, and microorganisms. Alginate has also been investigated for in situ gels, injectable hydrogels, 3D bioprinting bioinks, and tissue-engineering scaffolds, where gelation behavior, mechanical stability, and cytocompatibility are essential.

#### 2.3.3. Readiness Appraisal

Among the three polymers, alginate appears relatively advanced for selected pharmaceutical and biomedical applications. Its mild calcium-mediated gelation and broad use in hydrogels, beads, wound dressings, and encapsulation systems make it one of the more practically mature marine-derived polymers. However, apparent application maturity should not be equated with universal readiness. Alginate performance remains strongly dependent on M/G ratio, block distribution, molecular weight, ionic environment, and impurity profile. Therefore, alginate may be considered relatively close to readiness for selected applications, but only when polymer grade, composition, crosslinking conditions, and safety specifications are clearly defined [39,40].

### 2.4. Carrageenan

#### 2.4.1. Source, Extraction, Purification, and Critical Material Attributes

As a thickener, stabilizer, gelling agent and matrix forming polymer, carrageenan has long been known in many systems. Its activity is relevant in pharmaceutical aspects mainly for rheological regulation and as structure formation (in liquid, semi-solid or some solid/capsules systems) [14,41]. Variations between carrageenan types, particularly κ- and ι-carrageenan, provide a range of gelling behaviors that can be useful in formulation design [42].

At first glance, carrageenan may appear less chemically versatile than chitosan because its pharmaceutical use is often framed around thickening, gelation, and matrix-forming properties. However, this view underestimates its formulation-dependent behavior. Carrageenan performance is strongly governed by subtype, sulfate number and position, ionic environment, polymer concentration, and system composition, all of which influence gelation, rheology, syneresis, swelling, and controlled-release behavior. As a result, viscosity, elasticity, gel strength, and matrix behavior may vary substantially depending on formulation context [43,44].

Carrageenan thus undermines the presumption that excipient readiness is achieved more readily in cases where the intended function seems simpler. Even if a polymer is not described as a biologically active or highly interactive carrier, it then becomes only problematic to treat as broadly reliable if its rheological and structural behavior are strongly subtype- or environment-dependent. Carrageenan serves to extend this genial point by pointing out that utility in a specific formulation role must not be conflated with preparedness as a broadly transferable excipient [44,45].

Extraction and purification processes can influence carrageenan type, sulfate content, molecular weight distribution, residual salts, ash content, trace metals, and the presence of degraded low-molecular-weight fractions. This is important because degraded carrageenan fractions may raise toxicological concerns distinct from those associated with high-molecular-weight formulation-grade carrageenan. Therefore, pharmaceutical use requires type-specific characterization and careful control of molecular weight distribution, impurity profile, ionic composition, and degradation products [46,47].

#### 2.4.2. Pharmaceutical Applications

Carrageenan is relevant to pharmaceutical formulations because of its gel-forming, thickening, stabilizing, viscosity-enhancing, and matrix-forming properties. In oral dosage forms, carrageenan can function as a matrix-forming polymer, tablet binder, or viscosity modifier. Its hydration and gelation behavior may support oral matrices and formulations requiring structural integrity. In semisolid and topical formulations, carrageenan can contribute to gel consistency, spreadability, and physical stability. It may also be used in buccal films and other mucosal systems where film formation, hydration, and residence time are relevant. In suspension or emulsion-like systems, carrageenan may contribute to stabilization by increasing viscosity and reducing phase separation [42,48].

These applications show that carrageenan has value as a function-specific excipient, particularly where rheology control, stabilization, gel formation, or matrix formation is required. However, its utility remains dependent on carrageenan type, ionic environment, concentration, and formulation composition. Therefore, carrageenan should not be treated as a universal substitute for animal-derived or conventional excipients, but as a polymer whose pharmaceutical value is strongest in defined formulation roles [42,49].

#### 2.4.3. Readiness Appraisal

Carrageenan challenges the assumption that excipient readiness is achieved more easily when the intended function appears simpler. Even when a polymer is not described as a biologically active or highly interactive carrier, it may still be difficult to treat as broadly reliable if its rheological and structural behavior are strongly subtype- or environment-dependent. Carrageenan is therefore best viewed as a promising but formulation-dependent marine-derived excipient candidate. Its readiness is strongest in applications where thickening, gelation, stabilization, film formation, or matrix formation are the primary functions. However, broader pharmaceutical readiness requires type-specific specifications, molecular weight control, impurity profiling, and route-specific safety evaluation [44,45].

**Figure 1:**
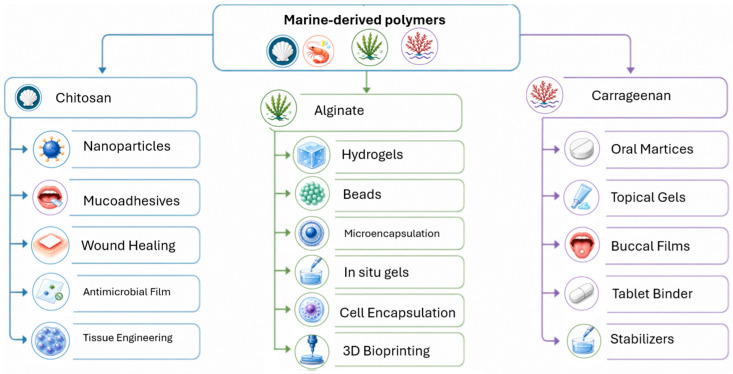
Pharmaceutical applications of marine-derived polymers as excipients and biomaterials.

Figure 1 summarizes representative pharmaceutical applications of chitosan, alginate, and carrageenan. Chitosan is mainly associated with nanoparticles, mucoadhesive delivery, wound healing, antimicrobial films, and tissue engineering. Alginate is widely investigated for hydrogels, beads, microencapsulation, in situ gels, cell encapsulation, and 3D bioprinting bioinks. Carrageenan is relevant to oral matrices, topical gels, buccal films, stabilizers, and tablet binders. These applications illustrate the function-specific potential of marine-derived polymers while emphasizing that application diversity does not automatically indicate general pharmaceutical excipient readiness.

### 2.5. Comparative Readiness of Chitosan, Alginate, and Carrageenan

Chitosan, alginate and carrageenan were chosen not to imply that any of the marine-derived polymers discussed are fully developed phases in a transition to replace conventional excipients, rather they highlight various degrees of translational distance between laboratory function and reliable pharmaceutical application. The value of their inclusion in this review is not just due to how popular they have become, but rather each illustrates a different sort of barrier that remains an impediment to more consistent excipient use.

Chitosan highlights a material whose pharmaceutical appeal is closely tied to structural responsiveness, yet whose performance is highly dependent on attributes such as molecular weight, degree of deacetylation, and charge-related behavior. Alginate, by contrast, appears closer to application because its gel-forming utility is easy to demonstrate and widely exploited, but its apparent maturity can obscure the extent to which formulation performance remains composition-dependent. Carrageenan illustrates a different problem again: even when the intended function seems comparatively simple, such as rheological regulation or matrix formation, performance may still remain strongly shaped by subtype, ionic environment, and overall system composition [26,50,51].

Collectively, these three polymers illustrate that marine-derived materials face no one-size-fits-all readiness problem. Instead, they disclose alternative paths on which useful biomaterials continue to be hard and challenging to translate into more reliable excipients: chitosan through condition sensitivity, alginate through composition-dependent normalization, and carrageenan through rheological context dependence. They are informative as test cases precisely for this reason. Their ongoing limitations imply that the true obstacle is not a dearth of exciting marine candidates, but rather the challenge converting biologically derived inputs with context-sensitive behavior into excipients capable of operating in a sufficiently repeatable, interpretable, and manufacturing-relevant manner [14,52].

While Figure 1 summarizes representative pharmaceutical applications, Table 1 provides a readiness-oriented comparison of the three polymers. This distinction is important because application diversity does not necessarily indicate excipient readiness. Instead, readiness depends on whether the material can be specified, controlled, reproduced, and qualified for a defined pharmaceutical function.

## 3. From Material Potential to Excipient Readiness

**Figure 2:**
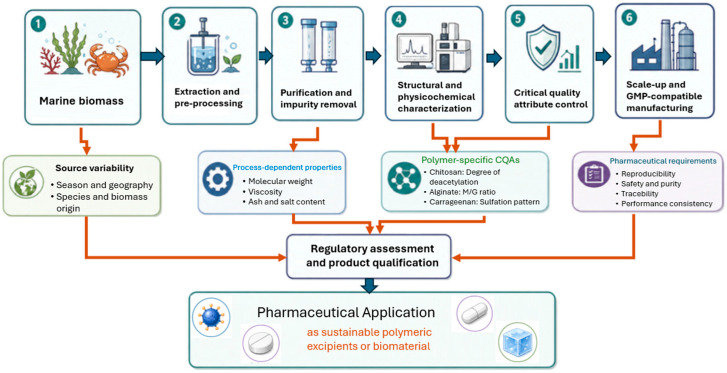
Translation pathway of marine polysaccharides from biomass to pharmaceutical-grade polymeric materials.

Using chitosan, alginate, and carrageenan as test cases, this section examines why marine-derived polymers with clear biomaterial value do not necessarily advance toward excipient readiness. In pharmaceutical development, usefulness as a biomaterial is not sufficient; a material must also demonstrate controllable attributes, reproducible performance, acceptable safety, manufacturability, and a realistic path to qualification [22]. This issue is particularly relevant for marine-derived polymers because their biological origin, extraction history, and structure-sensitive behavior can make critical material attributes difficult to stabilize [21]. Since such variability may affect dosage-form performance, stability, manufacturing consistency, and regulatory defensibility, the key question is whether these polymers can be controlled sufficiently to function as excipients rather than remain promising biomaterials [12]. Accordingly, Figure 2 provides a schematic overview of the translation pathway for marine polysaccharides, showing how biomass sourcing, extraction, purification, physicochemical characterization, safety assessment, quality control, and regulatory considerations collectively support their development into pharmaceutical-grade polymeric materials.

### 3.1. Source Variability and Extraction–Purification Burden

One of the biggest hurdles to marine-derived polymers is that this variability starts at the source. Material composition and structure can be influenced by species, provenance, harvest conditions, etc., and upstream biological variability can alter polymer composition and structure, with consequences for downstream pharmaceutical performance [20,60]. Source variability is therefore not an upstream inconvenience; it is often the first step in a chain of downstream uncertainty affecting CMAs, formulation performance, and qualification burden. The challenge is not only formulation performance but the generation of a suitably consistent starting material for pharmaceutical use. In chitosan, differences in chitin source and deacetylation conditions can generate substantial variation in degree of deacetylation, molecular weight, and solubility behavior. In alginate, mannuronate/guluronate composition and block distribution are central determinants of gelation and matrix properties [61,62]. In carrageenan, subtype and associated ionic response shape rheological behavior and gel formation [63,64].

Extraction and purification are designed to somewhat lessen this heterogeneity, but it cannot be eliminated fully. This complicates the process, and adds additional requirements with respect to the control of impurities, sensitivity to structure, loss of yield and cost instead [20,21]. These steps may enhance material suitability, but can also introduce further variability if processing conditions are not stringently regulated. In readiness terms, the burden of marine-derived polymers begins not at the formulation stage, but at the raw-material-to-intermediate-material transition itself.

### 3.2. Control of Critical Material Attributes

Among all barriers discussed in this review, control of CMAs is the central bottleneck because it determines whether a material can move from occasional functionality to reproducible pharmaceutical behavior. The solubility, mucoadhesion, and particle-forming performance of chitosan is affected by the degree of deacetylation and molecular weight [40,42,65]. Alginates with different M/G ratios and block structures exhibit differences in gel strength, matrix stability, and crosslinking response [40]. The elasticity, viscosity and gelation results also depend on the subtype of carrageenan used and the ionic conditions [42,66]. These parameters are not merely formulation details; they determine whether polymers with the same name, such as chitosan, alginate, or carrageenan, can produce comparable pharmaceutical performance across studies, manufacturing processes, and production batches.

That is why proof-of-concept performance is no longer sufficient. Because a polymer demonstrates one advantageous effect under a specific set of laboratory conditions does not mean that it can be treated as a controlled material. What counts is whether such an effect can be achieved within a clearly defined and interpretable space of attributes. Without that kind of control, performance is only half-decoupled from inherently unstable or poorly characterized inputs, and the material cannot be considered functionally uniform in any rigorous pharmaceutical sense [12].

The problem is compounded by inconsistent reporting. Even when key attributes are known to determine performance, they are not always disclosed or controlled in a standardized way. As a result, apparent agreement in polymer name does not guarantee agreement in material behavior. This undermines interpretability, limits reproducibility, and weakens any attempt to evaluate the material systematically.

### 3.3. Safety and Impurity Burden

Marine-derived polymers are often considered safe, natural, and biocompatible. Though these descriptors might provide some insight into their appeal, they are not valid reasons for pharmaceutical acceptance. A very common case in the pharmaceutical mode of development is the safety cannot be inferred from origin; instead, it requires adequate impurity control and specification discipline as well as process consistency. Marine-derived materials can retain residual proteins, salts or mineral matter, metal contaminants, endotoxins and other co-extracted or process-related impurities, depending on source and extraction history [12,21,67]. These factors may undermine, in itself, the toxicological acceptability, but also consistency of materials, stability of dosage forms and reliability of pharmaceutical performance [68].

As such, the impurity burden of marine-derived polymers needs to be considered at a minimum of three levels: source-associated residues; process-transferred contaminants or degradation products; and environmentally acquired contaminants with a biological origin [21,42]. While the importance of this burden differs by material and process, its translational influence is large. Residual proteins or minerals can affect interaction behavior or later complicate standardization, incomplete purification can jeopardize consistency and environmental contamination risks can worsen analytical/qualification requirements [12,67].

This issue is particularly important because natural materials are sometimes assumed to be inherently benign, an assumption that is not acceptable in pharmaceutical excipient science. From the standpoint of readiness, safety is not an inherent property of marine origin. It is the outcome of purification robustness, impurity characterization, batch control, toxicological evaluation, and quality assurance. Thus, safety burden is not separate from excipient readiness; it is part of the same translational problem. Incomplete control over impurities simultaneously weakens consistency, manufacturability, and regulatory confidence. Accordingly, Table 2 summarizes the key safety assessment and impurity characterization considerations that should be addressed when evaluating marine-derived polymers for pharmaceutical excipient applications.

### 3.4. Formulation-Specific Performance Limitations

The fact that a marine-derived polymer offers a useful pharmaceutical function does not mean that it can broadly replace conventional excipients across dosage forms. Chitosan may be highly valuable for mucoadhesion, electrostatic interactions, and particulate-system design, but these advantages do not make it a general substitute for excipients that provide other performance mechanisms, such as thermoreversible gelation or broader process tolerance [82,83]. Alginate is attractive as an ionotropic hydrogel former, yet its performance remains highly dependent on composition and ionic conditions that may vary across formulations [39,84]. Carrageenan can function effectively as a rheology modifier and matrix-forming agent, but its usefulness depends on subtype, concentration, ionic environment, and formulation context [12,85,86].

These polymers have substantial pharmaceutical value, but their utility remains context-dependent. Their advantages arise from specific mechanisms and formulation settings rather than from a general capacity to replace established excipients. A recurring limitation in the marine biomaterials literature is the tendency to equate success in a specific function with general excipient competitiveness. Strong performance in a defined application does not necessarily translate into broader excipient readiness, which depends on reproducibility, controllability, manufacturability, and a credible basis for qualification.

Formulation appraisal should move beyond desirable properties alone. A polymer’s ability to form gels, stabilize matrices, or support encapsulation is meaningful only if these functions are reproducible, competitive, and robust under pharmaceutically realistic conditions relative to established excipients [12,87]. Marine-derived polymers should be viewed as function-specific excipients rather than universal replacements, with their value determined by the range of formulation contexts in which they can perform reliably.

### 3.5. Scale-Up, Regulatory Issues, and Process Economics

Even if a marine-derived polymer works well in formulations in the lab, that does not guarantee it will see use in practice. Translation is not limited to functional performance alone but also involves scalability with reproducibility in the manufacturing process, and cost-effectiveness that remains realistic when compared to incumbent excipients [14,22]. A polymer that looks good at bench scale may not look so promising if scale-up drives instability or process sensitivity, or an unbearable qualification burden.

This challenge is often masked when biomass abundance is used to represent feasibility. Nevertheless, an abundant raw material does not mean that a competitive pharmaceutical excipient would automatically be derived out of it; the costs relevant to create one go well beyond just source availability. They also include extraction, purification, fractionation, analytical characterization, impurity control, batch qualification and the effort involved in maintaining specifications [12,22]. The economic question is therefore not “Is the source abundant?” but “Can a pharmaceutically credible material be produced repeatedly and competitively from that source?”.

This adds another level of complexity in the regulatory space. Excipient candidates with components that have not yet been established well enough for pharmaceutical application must receive additional support regarding quality, safety, consistency, and manufacturing control before a serious candidacy can be implied [22,68]. The familiarity with regulation is therefore not just a passive historical advantage of conventional excipients, it is a substantive competition barrier for any new materials. In halal-relevant development, the difficulty of qualification expands even further since readiness is conditional on not only material identity but also process integrity, auxiliary substances, traceability, and controls of cross-contamination throughout the supply chain [88].

In combination, these factors indicate that excipient readiness is not the sole attribute of the polymer. This is from the integration of controls over sourcing, purification, attributes stability, safety assurance, manufacturability qualified effort and the economic realism. For marine-derived polymers, failure at any of these levels can slow or prevent adoption. The challenge, therefore, is cumulative rather than isolated and multiple translational constraints converge on the same defect core: transforming structurally and biologically heterogeneous starting materials into pharmaceutical inputs that are sufficiently consistent, controllable and defensible for practical implementation.

### 3.6. The Cumulative Nature of Translational Barriers

A critical insight from this discussion is that the barriers mentioned above should not be treated as separate checklist items. Instead, they function as interlocking systems of constraint. Variabilities due to the source imply higher purification requirements; complexities associated with purification can impact purity burden and the structure of the entity under study; instability in critical material attributes leads to loss of drug product formulation reproducibility and poor reproducibility challenges scalability, qualifiability, and regulatory acceptability. This readiness challenge is thus systemic rather than modular.

This also explains why marine-derived polymers may appear highly promising in isolated studies yet still progress slowly toward routine excipient use. A formulation might work, a gel could form or a delivery system exhibit attractive performance, but the underlying material may still be inadequately controlled for robust pharmaceutical use. The central translational problem is therefore not lack of function, but rather lack of reproducibly controlled pharmaceutical and biopharmaceutical manufacturing across the entire pathway from source to qualified material. On this basis, future development can be formulated as the pursuit of greater readiness rather than a search for more promise. Table 3 summarizes the major technical domains affecting the pharmaceutical excipient readiness of chitosan, alginate, and carrageenan.

## 4. Evaluation Framework and Development Direction

Regardless, across chitosan, alginate, and carrageenan, the common theme is that the problem has changed from a lack of proof-of-concept formulation studies to a standardized framework for assessing excipient readiness. This is evidenced by the recent literature which highlights translational deficiencies that persist across the field and include poor material attribute control, unverifiable structural characterization, long-term instability during processing/storage steps, limited scaling reproducibility, as well as agnostic separation between laboratory functionality from commercial pharmaceutical-quality materials [22,81,90]. In combination, these patterns suggest that the primary barrier is not a lack candidate materials, but rather an insufficient implementation-focused evaluation.

### 4.1. From Candidate Material to Readiness-Oriented Evaluation

As in the area of biopharmaceutical research, marine biomass abundance is no indication per se of pharmaceutical suitability; nor is multiple proof-of-concept success a bona fide marker for molecular translational maturity. The facie proximity of a marine polymer to excipient readiness is better evaluated by its ability to demonstrate well-controlled, critical material attributes, reproducibility in quality, sufficient impurity control, consistent formulation behavior and realistic manufacturing feasibility under pharmaceutically relevant conditions [81,91,92]. Thus, evaluation should not focus on the number of reported applications, but rather on the extent to which the same material can be characterized, reproducibly, and qualified consistently across batches, processes, and intended uses. In this framework, readiness is not synonymous with multifunctionality, but with material controllability for pharmaceutical use [81,93].

### 4.2. What the Three Materials Teach About Readiness

The three materials reviewed here offer different lessons, but these all converge on similar translational conclusions. The primary hurdle presented by chitosan, for instance, is that its pharmaceutical functions of highest interest (mucoadhesion, charge-mediated interactions, and particle formation) carry a high dependence on nuanced material characteristics and are poorly standardized over time. Even for alginate, proximity to application does not eliminate significant translational complexity: the nature of hydrogel/encapsulation systems continues to be dictated by composition, block distribution and ionic conditions that govern their gelation behavior [37,45,94]. For carrageenan, the challenge is other in nature: even apparently more straightforward functionalities like rheological regulation and matrix formation remain extremely conditioned by formulation context and use conditions, making them challenging to define as stable excipient functionalities over systems [45,90].

These three cases illustrate the limitations of each of these marine polymers, with a translational bottleneck unique to each natural polymer. Chitosan is restricted due to attribute sensitivity, alginate by structural and gelation dependence, while carrageenan is given constraints based on formulation context. So, the material value itself does not ensure its acceptance in an excipient context; it is important to understand key limitations for each candidate material.

### 4.3. Priorities for Future Research

If marine polymers are to move closer to being ready as excipients, future research will need to shift from application exploration to demonstrating material controllability under pharmaceutically relevant constraints. Key priorities include raw material standardization, mapping of process–attribute relationships, impurity control, storage stability, inter-batch consistency, and meaningful comparison with established excipients [19,51].

In this context, purification strategies should no longer be judged primarily by extraction yields or laboratory convenience, but by their ability to produce materials with interpretable, stable, and reproducible critical attributes [67]. Similarly, formulation studies will need to move beyond simply demonstrating that a delivery system can be manufactured and begin assessing whether the polymer can perform consistently and competitively with existing excipients under realistic development conditions. Without such a shift, marine polymers will likely continue to generate an extensive proof-of-concept literature, but remain slow to translate into practical excipient inputs [22,67].

### 4.4. A Practical Readiness Perspective for Future Development

The analysis in this review suggests that a readiness-oriented assessment needs to be formulated as a practical evaluation framework to assess how close marine polymers are to moving toward excipient status. At a minimum, this framework should assess five areas: (1) compositional stability resulting from the source material and extraction process; (2) measurability, interpretability, and controllability of critical material attributes between batches; (3) adequacy of impurity reduction and characterization; (4) functional reliability in the intended dosage-form role under realistic formulation and manufacturing conditions; and (5) feasibility of scale-up, qualification, and economic-regulatory justification [12,22,51,95]. If these questions cannot yet be answered conclusively, a material may still be scientifically promising, but not yet close enough to excipient readiness. Thus, this practical readiness perspective is useful for shifting the development focus from a repetitive narrative of potential to evidence of controllability, reproducibility, and applicability. Table 4 summarizes a practical readiness framework for future development of marine-derived pharmaceutical excipients.

### 4.5. Translational Bottlenecks in Stage-Gate Excipient Development

In summary, this analysis can be mapped to a stage-gate framework for new excipient development. It is not the presence or absence of pharmaceutical functionality that causes candidate materials to fail, but rather their inability to convert that functionality into a suitable mode of action and predictable activity. Implementing technology transfer to the manufacturing node, particularly during the mid-translational stage, is the most critical gate determining long-term success. At this point, variability, incomplete attribute control, and impurity or process sensitivity can hinder comparison and scale-up. This is consistent with current excipient QbD thinking, which recognizes that excipient variability impacts drug product quality and must be incorporated into development and control strategies [12,13,95].

This framework also has relevance beyond marine-derived polymers. It identifies a general translational inflection point in novel excipient development: the stage at which an interesting material must begin to behave like a dependable excipient. At that point, attributes must be defined, measured, controlled, and linked to reproducible formulation performance under conditions meaningful to manufacturing and qualification.

AI-assisted approaches are now emerging in this space, but they serve a more limited role compared to drug discovery. Recent advances include the development of machine-learning models for drug–excipient compatibility prediction, AI formulation design tools and data-driven excipients recommendation platforms. However, implementation has been restricted by fragmented datasets; inconsistent descriptor reporting; lack of standardization; and persistent interpretability and regulatory acceptance challenges. At this stage, AI is best leveraged as a decision-support layer on top of screening, design and prioritization activities but will not replace necessary experimental and quality-building work needed to qualify an excipient [97,98,99].

## 5. Conclusions

This review shows that the main barrier to developing marine-derived polymers as pharmaceutical excipients is not the lack of candidate materials, but the difficulty of converting biologically variable renewable raw materials into standardized, safe, reproducible, and manufacturable pharmaceutical-grade excipients. Chitosan, alginate, and carrageenan have demonstrated important pharmaceutical functions in drug delivery systems, hydrogels, films, beads, matrices, wound dressings, and encapsulation platforms. However, reported functionality alone does not indicate excipient readiness.

Their readiness depends on the control of source variability, extraction and purification conditions, critical material attributes, impurity profiles, toxicological safety, formulation performance, and scale-up feasibility. Chitosan is strongly influenced by molecular weight and degree of deacetylation; alginate by M/G ratio, block structure, and ionic conditions; and carrageenan by subtype, sulfation pattern, molecular weight distribution, and formulation environment. These attributes determine whether polymers with the same nominal identity can provide reproducible pharmaceutical performance across batches, processes, and applications.

Therefore, marine-derived polymers should be evaluated as application-specific excipient candidates rather than universal replacements for established animal-derived or conventional excipients. Future development should move beyond proof-of-concept applications toward quantitative characterization, impurity profiling, toxicological evaluation, batch-to-batch comparison, process–attribute mapping, and meaningful comparison with existing excipients. By shifting the discussion from material promise to excipient readiness, this review provides a more practical basis for advancing sustainable marine-derived polymers toward pharmaceutical use.

## Figures and Tables

**Table 1:** The readiness appraisal of chitosan, alginate, and carrageenan as marine-derived excipient candidates.

Polymer	Strongest Readiness Position	Key Attributes	Main Readiness Barriers	Readiness Interpretation	Refs.
Chitosan	Strongest in mucoadhesive, topical, wound-contact, antimicrobial, and particulate systems	Degree of deacetylation, molecular weight, viscosity, charge density, pH-dependent solubility, residual proteins, ash content	Grade-to-grade variability, pH-dependent behavior, source- and process-dependent molecular properties, impurity control	Promising but highly grade- and route-dependent; best considered a specification-sensitive excipient candidate	[53,54,55]
Alginate	Strongest in gel-based, bead-based, encapsulation, wound dressing, and cell-contact systems	M/G ratio, guluronic acid block content, molecular weight, viscosity, ionic composition, residual salts, heavy metals, endotoxin burden	Composition-sensitive gelation, crosslinking variability, ionic sensitivity, impurity and endotoxin control	Relatively advanced for selected applications, but not universally ready; readiness depends on defined grade, composition, and crosslinking conditions	[39,40,56]
Carrageenan	Strongest in rheology control, gelation, stabilization, film formation, and matrix-forming systems	Carrageenan subtype, sulfation pattern, molecular weight distribution, viscosity, ionic sensitivity, ash/salt content, degraded low-molecular-weight fractions	Subtype-dependent behavior, formulation-dependent rheology, ionic sensitivity, need to distinguish high-molecular-weight grades from degraded fractions	Promising but formulation-dependent; best treated as a type-specific and route-specific excipient candidate	[57,58,59]

**Table 2:** Safety assessment and impurity characterization considerations for marine-derived pharmaceutical polymers.

Impurity or Safety Concern	Possible Source	Potential Pharmaceutical Relevance	Characterization or Evaluation	Refs.
Residual proteins	Incomplete deproteinization of marine biomass, especially crustacean-derived chitin/chitosan	Allergenicity, immunogenicity, batch variability	Protein assay, allergen screening, immunogenicity assessment	[55,69]
Ash and inorganic salts	Marine biomass, incomplete purification, ion-exchange or extraction residues	Altered viscosity, gelation behavior, ionic response, stability	Ash content, ion chromatography, elemental analysis	[57,70]
Heavy metals	Marine environmental contamination, seaweed or biomass source	Toxicological concern, regulatory burden	Inductively Coupled Plasma–Mass Spectrometry (ICP-MS) or atomic absorption spectroscopy	[71,72]
Endotoxins	Microbial contamination during harvesting, extraction, purification, or storage	Pyrogenicity and inflammatory response, especially for wound- or cell-contact applications	Endotoxin testing, microbial control	[73,74]
Microbial contamination	Biomass handling, water quality, drying, storage	Product safety and stability risk	Microbial limit testing	[75,76]
Residual solvents or reagents	Extraction, purification, deacetylation, precipitation, washing	Process-related toxicity and regulatory concern	Gas Chromatography (GC), High-Performance Liquid Chromatography (HPLC), residual solvent analysis	[21,77]
Low-molecular-weight degradation products	Harsh extraction, depolymerization, thermal or chemical degradation	Altered toxicity, viscosity, release behavior, or biological response	Gel Permeation Chromatography (GPC)/Size Exclusion Chromatography (SEC), viscosity measurement, degradation profiling	[78,79]
Structural variability	Source species, extraction conditions, purification history	Inconsistent drug release, gelation, mucoadhesion, or rheology	Nuclear Magnetic Resonance (NMR), Fourier Transform Infrared (FTIR), GPC/SEC, viscosity analysis, composition-specific assays	[80,81]

**Table 3:** Technical review of barriers limiting the pharmaceutical readiness of chitosan, alginate, and carrageenan.

Technical Domain	Why It Matters for Excipient Readiness	Chitosan	Alginate	Carrageenan	Implication for Pharmaceutical Readiness	Refs.
Source consistency and composition	Excipients must show predictable quality and performance across batches	Influenced by the chitin source, the degree of deacetylation, the molecular weight, and deacetylation process	Influenced by the mannuronate/guluronate ratio, block distribution, and algal source	Influenced by κ/ι/λ type, sulfate distribution, and ionic conditions	Biological variability increases the risk of batch-to-batch performance differences	[12,21,40]
Extraction and purification burden	Pharmaceutical excipients require clean, stable, and reproducible materials	Requires deproteinization, demineralization, and controlled deacetylation	Gel-relevant structure must be preserved during isolation	Fractionation and composition control are important for gel/rheological behavior	Abundant biomass does not automatically yield inexpensive or easily standardized pharmaceutical-grade material	[12,21,89]
Critical material attributes (CMAs)	CMAs directly affect processability and product performance	Degree of deacetylation and molecular weight influence mucoadhesion, solubility, and particle formation	M/G ratio influences gel strength and matrix stability	Carrageenan type and ionic environment influence elasticity, viscosity, and gel strength	Materials remain difficult to classify as ready when CMAs cannot be tightly controlled	[40,42,65]
Fit with dosage-form function	Excipients are judged by formulation-specific roles, not by general promise	Strong for mucoadhesion and particulate systems, but not universal	Strong for hydrogels and encapsulation, but highly context-dependent	Strong for rheology and matrix formation, but not universal	These polymers are better understood as function-specific excipients than broad replacements	[83,84,87]
Safety and impurities	Pharmaceutical quality requires impurity control and safety assurance	Risk of residual proteins/minerals and high need for preparation standardization	Final quality depends strongly on process control and impurity management	Characterization and quality control are required; natural origin alone is insufficient	“Natural” does not automatically mean pharmaceutical-grade safe	[12,21]
Scale-up, regulation, and economics	New materials are difficult to adopt if the total cost is high and the use volume remains low	Promising, but cost-effective industrialization remains challenging	Useful for specific applications, but still requires strict quality control	Exploration is increasing, but broad competitiveness is not yet established	Economic barriers arise from total processing cost, qualification burden, and lack of scale	[12,14]

**Table 4:** Practical readiness framework for future development of marine-derived pharmaceutical excipients.

Readiness Area	Key Question	Evidence Required	Relevance to Marine-Derived Polymers	Refs.
Source and compositional stability	Can the source material produce consistent polymer composition across batches?	Defined source, species, harvest or supply conditions, compositional profiling, inter-batch comparison	Addresses variability arising from marine biomass, species, geography, season, and extraction history	[57,80]
Critical material attribute control	Are the attributes that determine performance measurable and controllable?	Molecular weight, viscosity, degree of deacetylation, M/G ratio, sulfation pattern, ionic composition, gelation or rheology data	Links polymer structure to pharmaceutical performance and reproducibility	[21,80]
Impurity and toxicological profile	Are impurities reduced, characterized, and toxicologically acceptable?	Residual protein, ash, salts, heavy metals, endotoxins, microbial quality, degradation products, route-specific safety testing	Prevents reliance on natural origin as a substitute for pharmaceutical safety assessment	[40,55]
Functional reliability	Can the material perform its intended excipient role reproducibly?	Repeated formulation performance, stability data, release behavior, rheology, gel strength, mucoadhesion, or encapsulation performance	Distinguishes function-specific success from broad excipient readiness	[53,56]
Manufacturing and qualification feasibility	Can the material be produced and qualified under realistic pharmaceutical conditions?	Scalable purification, process controls, batch specifications, GMP-compatible manufacturing, regulatory documentation, cost evaluation	Determines whether promising laboratory materials can become dependable pharmaceutical excipient inputs	[21,96]

## Data Availability

No new data were created or analyzed in this study.
